# Supplementary material for: Single and Repeated Exposure to Cannabidiol Differently Modulate BDNF Expression and Signaling in the Cortico-Striatal Brain Network
Source: Biomedicines. 2022 Aug 1;10(8):1853. doi: 10.3390/biomedicines10081853 (PMC9405391; doi:10.3390/biomedicines10081853)
Supplement: Supplementary file 1 [file biomedicines-10-01853-s001.zip › biomedicines-1830400-supplementary.pdf]

Supplementary Table S1. Normality residuals calculation on data relative to CBD levels measured in the plasma and mPFC of saline and CBD treated rats with a single and repeated administration of CBD at the doses of 5, 15, 30 mg/kg represented in figure 1 and, 2.

CBD levels

Single treatment

	Normality of Residuals<br>Test name	Statistics	P value	Passed normality test (alpha=0,05)?	P value summary
**Plasma**	Shapiro-Wilk (W)	0,8971	0,0187	No	*
	Kolmogorov-Smirnov (distance)	0,1926	0,0216	No	*
**mPFC**	Shapiro-Wilk (W)	0,9111	0,0431	No	*
	Kolmogorov-Smirnov (distance)	0,1957	0,0226	No	*

Repeated treatment

**Plasma**	Shapiro-Wilk (W)	0,9352	0,1271	Yes	ns
	Kolmogorov-Smirnov (distance)	0,1704	0,0697	Yes	ns
**mPFC**	Shapiro-Wilk (W)	0,8050	0,0004	No	***
	Kolmogorov-Smirnov (distance)	0,2136	0,0060	No	**

Supplementary Table S2. Normality residuals calculation on data relative to the mRNA and protein levels measured in the mPFC of saline and CBD treated rats with a single administration of CBD at the doses of 5, 15, 30 mg/kg represented in figure 3, 4, 5.

Prefrontal cortex	Normality of Residuals<br>Test name	Statistics	P value	Passed normality test (alpha=0,05)?	P value summary
Total bdnf	Shapiro-Wilk (W)	0,9604	0,2986	Yes	ns
	Kolmogorov-Smirnov (distance)	0,1212	0,1000	Yes	ns
Bdnf exon IV	Shapiro-Wilk (W)	0,9152	0,0177	No	*
	Kolmogorov-Smirnov (distance)	0,1666	0,0282	No	*
Bdnf exon VI	Shapiro-Wilk (W)	0,9821	0,8679	Yes	ns
	Kolmogorov-Smirnov (distance)	0,09452	0,1000	Yes	ns
**mBDNF**	Shapiro-Wilk (W)	0,9807	0,8309	Yes	ns
	Kolmogorov-Smirnov (distance)	0,1037	0,1000	Yes	ns
**pTrkB**	Shapiro-Wilk (W)	0,9406	0,0779	Yes	ns
	Kolmogorov-Smirnov (distance)	0,1535	0,0532	Yes	ns
**TrkB**	Shapiro-Wilk (W)	0,9070	0,0094	No	**
	Kolmogorov-Smirnov (distance)	0,1513	0,0603	Yes	ns
**pTrkB/TrkB**	Shapiro-Wilk (W)	0,8956	0,0048	No	**
	Kolmogorov-Smirnov (distance)	0,1882	0,0055	No	**
**pAkt**	Shapiro-Wilk (W)	0,9560	0,2125	Yes	ns
	Kolmogorov-Smirnov (distance)	0,1470	0,0766	Yes	ns
**Akt**	Shapiro-Wilk (W)	0,8804	0,0020	No	**
	Kolmogorov-Smirnov (distance)	0,1196	0,1000	Yes	ns
**pAkt/Akt**	Shapiro-Wilk (W)	0,9362	0,0583	Yes	ns
	Kolmogorov-Smirnov (distance)	0,1224	0,1000	Yes	ns
**pERK2**	Shapiro-Wilk (W)	0,7852	<0,0001	No	****
	Kolmogorov-Smirnov (distance)	0,1809	0,0093	No	**
**ERK2**	Shapiro-Wilk (W)	0,9093	0,0108	No	*
	Kolmogorov-Smirnov (distance)	0,09915	0,1000	Yes	ns
**pERK2/ERK2**	Shapiro-Wilk (W)	0,9294	0,0378	No	*
	Kolmogorov-Smirnov (distance)	0,1179	0,1000	Yes	ns

Supplementary Table S3. Normality residuals calculation on data relative to the mRNA and protein levels measured in the mPFC of saline and CBD treated rats with repeated administration of CBD at the dose of 30 mg/kg represented in figure 6, 7, 8.

Prefrontal cortex	Normality of Residuals				
	Test name	Statistics	P value	Passed normality test (alpha=0,05)?	P value summary
Total bdnf	Shapiro-Wilk (W)	0,9794	0,7944	Yes	ns
	Kolmogorov-Smirnov (distance)	0,1170	0,1000	Yes	ns
Bdnf exon IV	Shapiro-Wilk (W)	0,9831	0,8838	Yes	ns
	Kolmogorov-Smirnov (distance)	0,09237	0,1000	Yes	ns
Bdnf exon VI	Shapiro-Wilk (W)	0,9678	0,4617	Yes	ns
	Kolmogorov-Smirnov (distance)	0,1384	0,1000	Yes	ns
mBDNF	Shapiro-Wilk (W)	0,9786	0,9516	Yes	ns
	Kolmogorov-Smirnov (distance)	0,1488	0,1000	Yes	ns
pTrkB	Shapiro-Wilk (W)	0,8782	0,0364	No	*
	Kolmogorov-Smirnov (distance)	0,2189	0,0390	No	*
TrkB	Shapiro-Wilk (W)	0,8747	0,0322	No	*
	Kolmogorov-Smirnov (distance)	0,1448	0,1000	Yes	ns
pTrkB/TrkB	Shapiro-Wilk (W)	0,9710	0,8543	Yes	ns
	Kolmogorov-Smirnov (distance)	0,1295	0,1000	Yes	ns
pAkt	Shapiro-Wilk (W)	0,9694	0,8291	Yes	ns
	Kolmogorov-Smirnov (distance)	0,1286	0,1000	Yes	ns
Akt	Shapiro-Wilk (W)	0,9661	0,7713	Yes	ns
	Kolmogorov-Smirnov (distance)	0,1358	0,1000	Yes	ns
pAkt/Akt	Shapiro-Wilk (W)	0,9172	0,1522	Yes	ns
	Kolmogorov-Smirnov (distance)	0,1921	0,1000	Yes	ns
pERK2	Shapiro-Wilk (W)	0,9784	0,9497	Yes	ns
	Kolmogorov-Smirnov (distance)	0,1317	0,1000	Yes	ns
ERK2	Shapiro-Wilk (W)	0,8869	0,0498	No	*
	Kolmogorov-Smirnov (distance)	0,2092	0,0596	Yes	ns
pERK2/ERK2	Shapiro-Wilk (W)	0,9820	0,9772	Yes	ns
	Kolmogorov-Smirnov (distance)	0,1241	0,1000	Yes	ns

Supplementary Table S4. Normality residuals calculation on data relative to the mRNA and protein levels measured in the striatum of saline and CBD treated rats with repeated administration of CBD at the dose of 30 mg/kg represented in figure 9, 10.

Striatum	Normality of Residuals				
	Test name	Statistics	P value	Passed normality test (alpha=0,05)?	P value summary
mBDNF	Shapiro-Wilk (W)	0,8777	0,0357	No	*
	Kolmogorov-Smirnov (distance)	0,1716	0,1000	Yes	ns
pTrkB	Shapiro-Wilk (W)	0,8251	0,0079	No	**
	Kolmogorov-Smirnov (distance)	0,2340	0,0265	No	*
TrkB	Shapiro-Wilk (W)	0,8998	0,0798	Yes	ns
	Kolmogorov-Smirnov (distance)	0,2049	0,0712	Yes	ns
pTrkB/TrkB	Shapiro-Wilk (W)	0,8846	0,0555	Yes	ns
	Kolmogorov-Smirnov (distance)	0,2386	0,0214	No	*
pAkt	Shapiro-Wilk (W)	0,9268	0,2166	Yes	ns
	Kolmogorov-Smirnov (distance)	0,1914	0,1000	Yes	ns
Akt	Shapiro-Wilk (W)	0,9133	0,1314	Yes	ns
	Kolmogorov-Smirnov (distance)	0,2060	0,0679	Yes	ns
pAkt/Akt	Shapiro-Wilk (W)	0,9080	0,1079	Yes	ns
	Kolmogorov-Smirnov (distance)	0,1518	0,1000	Yes	ns
pERK2	Shapiro-Wilk (W)	0,9693	0,8266	Yes	ns
	Kolmogorov-Smirnov (distance)	0,09494	0,1000	Yes	ns
ERK2	Shapiro-Wilk (W)	0,9617	0,6920	Yes	ns
	Kolmogorov-Smirnov (distance)	0,1371	0,1000	Yes	ns
pERK2/ERK2	Shapiro-Wilk (W)	0,8779	0,0360	No	*
	Kolmogorov-Smirnov (distance)	0,1795	0,1000	Yes	ns
